# Thiamine administration and in-hospital mortality in patients with traumatic brain injury: analysis of the MIMIC-IV database

**DOI:** 10.3389/fneur.2025.1448439

**Published:** 2025-04-24

**Authors:** Shuangqi Gao, Zhendan Zhu, Wenhan Zheng

**Affiliations:** Department of Neurosurgery, The Third Affiliated Hospital of Sun Yat-sen University, Guangzhou, China

**Keywords:** thiamine, traumatic brain injury, in-hospital mortality, intensive care unit, Medical Information Mart for Intensive Care IV

## Abstract

**Aim:**

Several studies have suggested the favorable impact of thiamine administration on the prognosis of diseases. However, the value of thiamine in patients with traumatic brain injury (TBI) admitted to the intensive care unit (ICU) remains unclear. The aim of this study was to investigate the association between the between thiamine administration and in-hospital mortality in TBI patients.

**Methods:**

A cohort of 1,755 individuals diagnosed with TBI from the Medical Information Mart for Intensive Care IV database were included in this retrospective cohort study. Thiamine administration is determined by the patient’s usage during their stay in the ICU. The primary outcome was in-hospital mortality. Univariable and multivariable Cox regression analysis were used to investigate the relationship between thiamine administration and in-hospital mortality of patients with TBI. Subgroup analysis was also performed to determine if this association differed for subgroups classified using different variables including age (<65 years and ≥65 years), gender (male and female), and the severity of TBI (mild, moderate, and severe).

**Results:**

The median follow-up time was 6.77 (3.98, 12.94) days, and the in-hospital mortality rate for the population was approximately 14.1%. In the univariable Cox regression analysis, thiamine administration was significantly associated with the reduced risk of in-hospital mortality in TBI patients admitted to the ICU. performing the multivariable Cox regression analysis, the observed association of thiamine administration and in-hospital mortality remained significant, with the hazard ratios (HR) of 0.66 [95% confidence interval (CI) = 0.45–0.98]. In the subgroup analysis, the results demonstrated that thiamine administration resulted in a decreased risk of in-hospital mortality among TBI patients who aged 65 years or older (HR = 0.36, 95% CI: 0.19–0.69), as well as male individuals (HR = 0.36, 95% CI: 0.17–0.80) and those with severe TBI (HR = 0.16, 95% CI: 0.04–0.57).

**Conclusion:**

Thiamine administration may reduce in-hospital mortality for patients with TBI admitted to the ICU.

## Introduction

Traumatic brain injury (TBI) is regarded as an acquired insult to the brain resulting from external mechanical force, which might lead to potential temporary or permanent impairment (1, 2). The research findings indicate that TBI has been a prominent contributor to global disability, morbidity, and mortality (3). High mortality rate of TBI is attributed not only to the severity of the initial brain injury, but also to systemic complications arising as a result of the brain injury (4, 5). Despite the gradual improvement and standardization of treatment options, the mortality associated with TBI remains a cause for concern, Therefore, exploring the new therapeutic approaches is crucial for improving the prognosis of TBI patients.

Thiamine is an essential water-soluble vitamin that plays a crucial role in various physiological processes (6). Thiamine deficiency may result in alterations in neurotransmitters, lactic acidosis, apoptosis, activation of oxidative stress response, inflammation, as well as dysfunction of the blood–brain barrier (7, 8). In the pathophysiology of TBI, the increasing of free radical and reactive oxygen species following the injury results in oxidative stress and subsequent secondary neurotoxicity (9). An animal experiment demonstrated that thiamine has the potential to effectively ameliorate mitochondrial damage and neuritis in rats with TBI (10). A retrospective cohort analysis revealed that thiamine was linked with decreased risk of in-hospital mortality among heart failure patients admitted to the intensive care unit (ICU) (11). Early thiamine administration was found to have an improvement in short-term survival outcomes for critically ill patients with acute kidney injury (AKI) (12). In addition, the study conducted by Yue et al. also highlighted a significant reduction in the risk of in-hospital, 30-day, and 90-day mortality among myocardial infarction patients receiving thiamine compared to those not receiving thiamine (13). These studies findings suggested the favorable impact of thiamine administration on the prognosis of diseases. However, the existing studies does not provide any evidence regarding the potential effect of thiamine in enhancing outcomes among critically ill individuals suffering from TBI.

Therefore, this study intends to investigate the association between thiamine administration and the risk of in-hospital mortality in patients with TBI based on Medical Information Mart for Intensive Care (MIMIC)-IV database. The findings from this research might provide valuable insights for treatment decision-making and prognosis enhancement in TBI patients.

## Methods

### Study population

This retrospective cohort study obtained all data from the MIMIC-IV, an openly accessible and freely available critical care database (14). This database contained comprehensive clinical data of patients who underwent inpatient treatment at the Beth Israel Deaconess Medical Center (BIDMC) between 2008 and 2019, such as basic patient information, vital signs, laboratory indicators and survival data. We did not require patient consent or ethical approval as all patient privacy information in the database has been de-identified.

**Figure 1:**
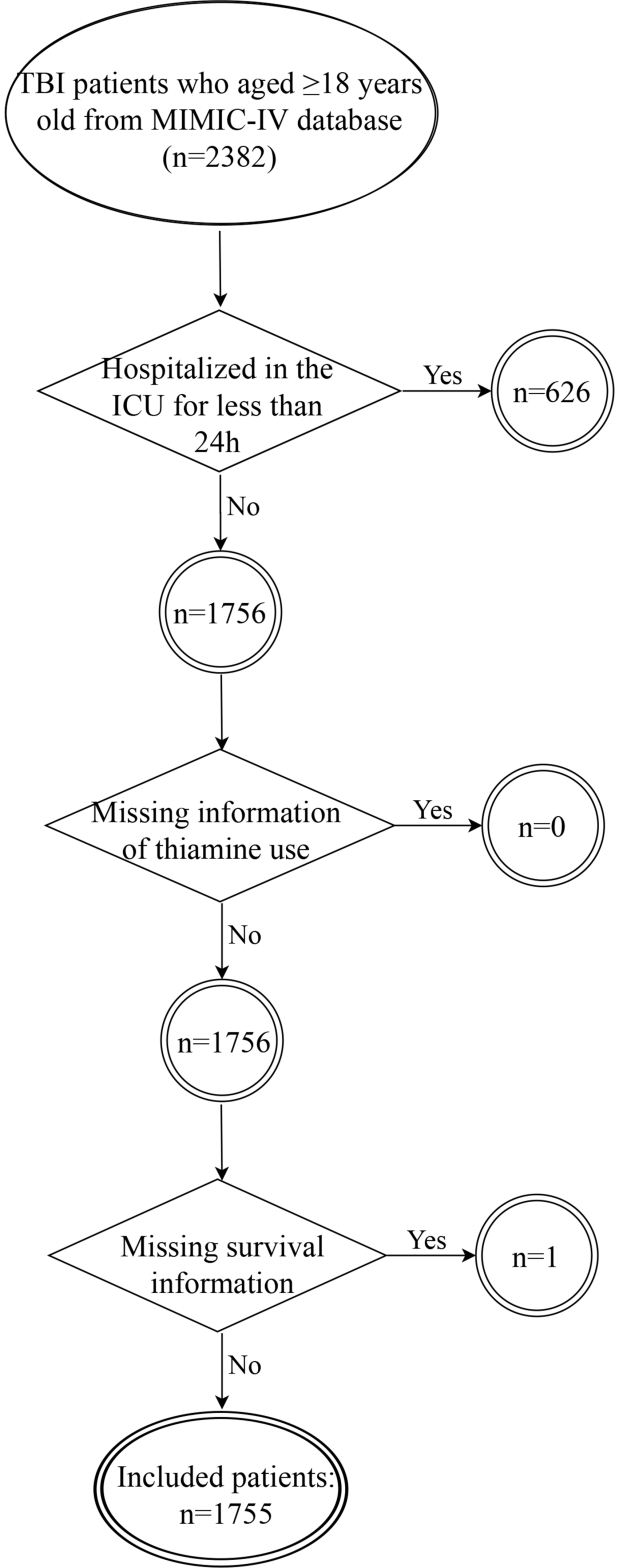
Flowchart for study participants enrolling.

We selected patients from the MIMIC-IV database based on the following criteria: (1) diagnosed as TBI at ICU admission [International Classification of Disease, Ninth (ICD-9: 85) and Tenth (ICD-10: S06) Versions]; (2) aged≥18 years old. The exclusion criteria for participation in the study were as follows: (1) hospitalized in the ICU for less than 24 h (*n* = 626); (2) missing information of thiamine use (*n* = 0); (3) missing survival information (*n* = 1). The flowchart for study participants enrolling is presented in Figure 1.

### Data collection

We extracted some important variables from the MIMIC-IV database: age (years), gender, race/ethnicity, insurance status, ICU type, urine output (mL), hypertension, deterioration of neurological function, acute kidney injury (AKI), heart rate (bpm), systolic blood pressure (SBP, mmHg), diastolic blood pressure (DBP, mmHg), respiratory rate (insp/min), temperature (°C), oxygen saturation (SpO_2_, %), Sequential Organ Failure Assessment (SOFA) score, Charlson comorbidity index (CCI), Glasgow Coma Score (GCS), Simplified Acute Physiology Score II (SAPSII), white blood cell (WBC, K/μL), platelet (K/μL), hemoglobin (g/dL), hematocrit (%), red cell distribution width (RDW, %), epidermal growth factor receptor (eGFR, mL/min/1.73m^2^), blood urea nitrogen (BUN, mg/dL), glucose (mg/dL), calcium (mg/dL), sodium (mEq/L), potassium (mEq/L), chloride (mEq/L), vasopressor, mechanical ventilation, mannitol, diuretic, surgery, and thiamine. Deterioration of neurological function was defined as a decline of ≥2 points in the GCS during the ICU period (15). Thiamine administration is determined by the patient’s usage during their stay in the ICU. The primary outcome of the present study was in-hospital mortality. For patients with multiple ICU admissions, only clinical data from the first ICU admission were utilized. Variables with more than 10% missing values were excluded from the study. Conversely, these variables with less than 10% missing values were imputed using multiple interpolation method. Supplementary Table 1 shows the sensitivity analysis on the data sets before and after interpolation.

### Statistical analysis

We reported the categorical variables as numbers and percentages. The normally distributed continuous variables were expressed as the means ± standard deviation (SD), the skewed distributed continuous variables as the median and interquartile range [M (Q₁, Q3)]. Among the groups, we compared categorical variables by Chi-squared test. Continuous variables were compared by T test (normally distributed) or Wilcoxon rank sum test (skewed distributed). Univariable and multivariable Cox regression analysis were used to investigate the relationship between thiamine administration and in-hospital mortality of patients with TBI, the results were expressed as hazard ratios (HR) and 95% confidence interval (CI). Model 1 was unadjusted (univariable Cox regression analysis). Model 2 (multivariable Cox regression analysis) adjusted for all confounding variables. *p* < 0.05 was considered statistically significant. Subgroup analysis was also performed to determine if this association differed for subgroups classified using different variables including age (<65 years and ≥ 65 years), gender (male and female), and the severity of TBI (mild, moderate, and severe). According to the GCS, patients with TBI can be categorized as mild (13–15 points), moderate (9–12 points), or severe (3–8 points) (16). In addition, Kaplan–Meier survival curves were employed to observe the relationship between thiamine administration and in-hospital mortality among patients with TBI. All statistical analyses were performed using SAS9.4 software.

## Results

### General characteristics

A total of 1,755 individuals diagnosed with TBI were included in this study, with an average age of 64.41 ± 20.26 years. Among them, 1,079 (61.48%) patients were men. The median follow-up time was 6.77 (3.98, 12.94) days, and the in-hospital mortality rate for the population was approximately 14.1%. Table 1 summarizes the comparison of characteristics between the survival (*n* = 1,538) and in-hospital mortality groups (*n* = 217). Compared with the survival group, the in-hospital mortality group showed older age (70.25 vs. 63.59 years, *p* < 0.001), higher prevalence of deterioration of neurological function (62.21% vs. 51.04%, *p* = 0.002) and AKI (80.65% vs. 54.68%, *p* < 0.001) but lower DBP, temperature, platelet, hematocrit, hemoglobin, eGFR, and calcium levels (*p* < 0.05). In addition, we also observed that the proportion of thiamine use in the in-hospital mortality group (16.13%) was lower than that of the survival group (21.65%^), although without statistical significance. The detailed population characteristics can be found in Table 1.

**Table 1:** Comparison of characteristics between the survival and in-hospital mortality groups.

Variables	Total (*n* = 1,755)	Survival group (*n* = 1,538)	In-hospital mortality group (*n* = 217)	Statistics	*p*
Age (years), Mean ± SD	64.41 ± 20.26	63.59 ± 20.37	70.25 ± 18.52	t = −4.56	<0.001
Gender, *n* (%)				χ^2^ = 1.572	0.210
Female	676 (38.52)	584 (37.97)	92 (42.40)		
Male	1,079 (61.48)	954 (62.03)	125 (57.60)		
Race, *n* (%)				χ^2^ = 18.978	<0.001
White	1,110 (63.25)	999 (64.95)	111 (51.15)		
Black	108 (6.15)	96 (6.24)	12 (5.53)		
Other	537 (30.60)	443 (28.80)	94 (43.32)		
Insurance, *n* (%)				χ^2^ = 14.976	<0.001
Medicaid	122 (6.95)	105 (6.83)	17 (7.83)		
Medicare	740 (42.17)	624 (40.57)	116 (53.46)		
Other	893 (50.88)	809 (52.60)	84 (38.71)		
ICU type, *n* (%)				χ^2^ = 3.394	0.335
MICU	102 (5.81)	84 (5.46)	18 (8.29)		
SICU	463 (26.38)	405 (26.33)	58 (26.73)		
TSICU	762 (43.42)	668 (43.43)	94 (43.32)		
Other	428 (24.39)	381 (24.77)	47 (21.66)		
Urine output (mL), M (Q_1_, Q_3_)	1632.00 (1070.00, 2310.00)	1631.00 (1085.00, 2290.00)	1670.00 (1005.00, 2425.00)	Z = 0.408	0.683
Hypertension, yes, *n* (%)	912 (51.97)	791 (51.43)	121 (55.76)	χ^2^ = 1.428	0.232
Deterioration of neurological function, yes, *n* (%)	920 (52.42)	785 (51.04)	135 (62.21)	χ^2^ = 9.516	0.002
AKI, yes, *n* (%)	1,016 (57.89)	841 (54.68)	175 (80.65)	χ^2^ = 52.588	<0.001
Heart rate (bpm), Mean ± SD	84.83 ± 18.53	84.56 ± 18.16	86.76 ± 20.91	t = −1.48	0.141
SBP (mmHg), Mean ± SD	132.73 ± 22.87	132.93 ± 22.44	131.29 ± 25.71	t = 0.90	0.371
DBP (mmHg), Mean ± SD	72.41 ± 16.98	72.77 ± 16.70	69.86 ± 18.68	t = 2.17	0.031
Respiratory rate (insp/min), Mean ± SD	18.52 ± 5.17	18.44 ± 5.08	19.08 ± 5.80	t = −1.55	0.122
Temperature (°C), Mean ± SD	36.80 ± 0.80	36.83 ± 0.73	36.56 ± 1.16	t = 3.33	<0.001
SPO_2_ (%), Mean ± SD	97.52 ± 3.62	97.47 ± 3.62	97.84 ± 3.61	t = −1.41	0.160
SOFA, M (Q_1_, Q_3_)	1.00 (0.00, 1.00)	0.00 (0.00, 1.00)	1.00 (0.00, 3.00)	Z = 6.719	<0.001
CCI, M (Q_1_, Q_3_)	1.00 (0.00, 2.00)	1.00 (0.00, 2.00)	1.00 (0.00, 3.00)	Z = 4.308	<0.001
GCS, M (Q_1_, Q_3_)	14.00 (13.00, 15.00)	14.00 (13.00, 15.00)	15.00 (9.00, 15.00)	Z = 0.147	0.883
SAPSII, M (Q_1_, Q_3_)	32.00 (25.00, 40.00)	31.00 (24.00, 38.00)	40.00 (33.00, 50.00)	Z = 11.853	<0.001
WBC (K/uL), M (Q_1_, Q_3_)	10.20 (7.50, 13.20)	10.00 (7.40, 13.00)	11.70 (8.80, 15.00)	Z = 4.905	<0.001
Platelet (K/uL), M (Q_1_, Q_3_)	189.00 (146.00, 242.00)	190.00 (150.00, 242.00)	174.00 (123.00, 224.00)	Z = -3.209	0.001
Hematocrit (%), Mean ± SD	34.20 ± 5.78	34.41 ± 5.66	32.65 ± 6.38	t = 3.86	<0.001
Hemoglobin (g/dL), Mean ± SD	11.40 ± 2.03	11.49 ± 2.00	10.80 ± 2.15	t = 4.66	<0.001
RDW (%), Mean ± SD	14.23 ± 1.90	14.09 ± 1.75	15.19 ± 2.55	t = −6.12	<0.001
eGFR (mL/min/1.73m^2^), Mean ± SD	95.20 ± 14.58	95.60 ± 14.66	92.34 ± 13.66	t = 3.09	0.002
BUN (mg/dL), M (Q_1_, Q_3_)	15.00 (11.00, 22.00)	15.00 (11.00, 21.00)	18.00 (14.00, 29.00)	Z = 6.330	<0.001
Glucose (mg/dL), M (Q_1_, Q_3_)	123.00 (102.00, 154.00)	121.00 (101.00, 149.00)	142.00 (114.00, 173.00)	Z = 6.291	<0.001
Calcium (mg/dL), Mean ± SD	8.09 ± 1.48	8.12 ± 1.46	7.88 ± 1.60	t = 2.23	0.026
Sodium (mEq/L), Mean ± SD	138.80 ± 4.90	138.74 ± 4.77	139.25 ± 5.69	t = −1.27	0.207
Potassium (mEq/L), Mean ± SD	4.07 ± 0.71	4.06 ± 0.70	4.14 ± 0.83	t = −1.27	0.207
Chloride (mEq/L), Mean ± SD	103.67 ± 5.70	103.61 ± 5.53	104.10 ± 6.73	t = −1.02	0.307
Vasopressors, yes, *n* (%)	228 (12.99)	157 (10.21)	71 (32.72)	χ^2^ = 85.252	<0.001
Mechanical ventilation, yes, *n* (%)	1,166 (66.44)	980 (63.72)	186 (85.71)	χ^2^ = 41.260	<0.001
Mannitol, yes, *n* (%)	49 (2.79)	25 (1.63)	24 (11.06)	χ^2^ = 62.366	<0.001
Diuretic, yes, *n* (%)	338 (19.26)	261 (16.97)	77 (35.48)	χ^2^ = 41.918	<0.001
Surgery, yes, *n* (%)	14 (0.80)	12 (0.78)	2 (0.92)	-	0.688
Thiamine, yes, *n* (%)	368 (20.97)	333 (21.65)	35 (16.13)	χ^2^ = 3.500	0.061
Follow-up-time (days), M (Q_1_, Q_3_)	6.77 (3.98, 12.94)	6.92 (4.21, 13.45)	5.15 (2.40, 10.46)	Z = -5.175	<0.001

ICU, intensive care unit; MICU, medical intensive care unit; SICU, surgical intensive care unit; TSICU, trauma and surgical intensive care unit; AKI, acute kidney injury; SBP, systolic blood pressure; DBP, diastolic blood pressure; SPO_2_, pulse oxygen saturation; SOFA, Sequential Organ Failure Assessment; CCI, Charlson comorbidity index; GCS, Glasgow Coma Score; SAPSII, Simplified Acute Physiology Score II; WBC, white blood cell; RDW, red cell distribution width; eGFR, estimated glomerular filtration rate; BUN, blood urea nitrogen.

### Thiamine administration and in-hospital mortality

**Figure 2:**
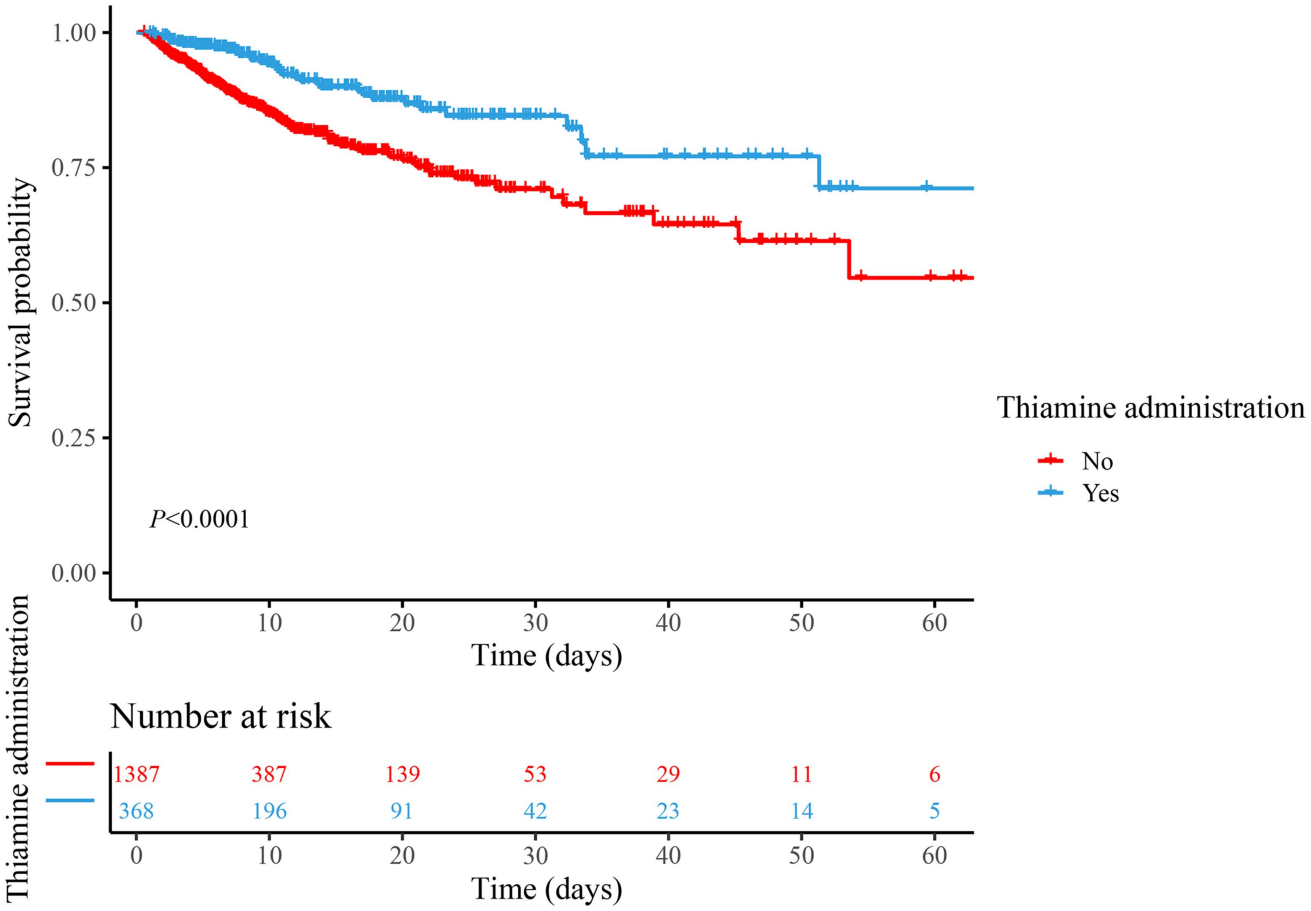
Comparison of survival between the thiamine group and the non-thiamine group using the Kaplan–Meier survival analysis in the cohort.

Univariable Cox regression identified 21 variables significantly linked to mortality (*p* < 0.05), including age (HR = 1.02, 95% CI: 1.02–1.03), AKI (HR = 1.96, 95% CI: 1.40–2.76), and SOFA score (HR = 1.21, 95% CI: 1.14–1.28), protective factors included hemoglobin (HR = 0.90, 95% CI: 0.84–0.95) and temperature (HR = 0.75, 95% CI: 0.67–0.84), as presented in Supplementary Table 2. These variables were subsequently adjusted in multivariable models to isolate the independent effect of thiamine on mortality. In both unadjusted and adjusted Cox regression analyses, we found that thiamine administration was significantly associated with the reduced risk of in-hospital mortality in TBI patients admitted to the ICU (Model 1: HR = 0.46, 95% CI: 0.32–0.67, *p* < 0.001; Model 2: HR = 0.66, 95% CI: 0.45–0.98, *p* = 0.037). Kaplan–Meier survival curves (Figure 2) suggested that the survival rate of thiamine administration group was significantly higher than that of the non-thiamine administration group (*p* < 0.001). The thiamine group demonstrated higher cumulative survival rates over the follow-up period (median: 6.77 days), aligning with regression results (Table 2).

**Table 2:** Thiamine administration and in-hospital mortality.

Variables	Model 1		Model 2	
	HR (95%CI)	*p*	HR (95%CI)	*p*
Thiamine administration				
No	Ref		Ref	
Yes	0.46 (0.32–0.67)	<0.001	0.66 (0.45–0.98)	0.037

Ref, reference; HR, hazard ratio; CI, confidence interval; Model 1: unadjusted; Model 2: adjusted age, gender, insurance status, urine output, acute kidney injury, diastolic blood pressure, temperature, Sequential Organ Failure Assessment, Charlson comorbidity index, Glasgow Coma Score, Simplified Acute Physiology Score II, white blood cell, hematocrit, hemoglobin, red cell distribution width, estimated glomerular filtration rate, blood urea nitrogen, glucose, sodium, vasopressor, mechanical ventilation, and mannitol.

### Subgroup analysis

We performed subgroup analysis based on age (<65 years and ≥ 65 years), gender (male and female), and the severity of TBI (mild, moderate, and severe) to assess the association between thiamine administration and in-hospital mortality in TBI patients admitted to the ICU (Table 3). After performing the multivariable Cox regression analysis, the results demonstrated that thiamine administration resulted in a decreased risk of in-hospital mortality among TBI patients who aged 65 years or older (HR = 0.36, 95% CI: 0.19–0.69, *p* = 0.002), as well as male individuals (HR = 0.36, 95% CI: 0.17–0.80, *p* = 0.012) and those with severe TBI (HR = 0.16, 95% CI: 0.04–0.57, *p* = 0.005).

**Table 3:** Subgroup analysis.

Variables	Age: <65		Age: ≥65	
	HR (95%CI)	*p*	HR (95%CI)	*p*
Thiamine administration				
No	Ref		Ref	
Yes	0.71 (0.39–1.27)	0.244	0.36 (0.19–0.69)	0.002

Variables	Gender: male		Gender: female	
	HR (95%CI)	*p*	HR (95%CI)	*p*
Thiamine administration				
No	Ref		Ref	
Yes	0.36 (0.17–0.80)	0.012	0.78 (0.49–1.24)	0.286

Variables	GCS: 13–15 points		GCS: 9–12 points		GCS: 3–8 points	
	HR (95%CI)	*p*	HR (95%CI)	*p*	HR (95%CI)	*p*
Thiamine administration						
No	Ref		Ref		Ref	
Yes	0.81 (0.50–1.29)	0.367	0.35 (0.11–1.14)	0.083	0.16 (0.04–0.57)	0.005

Ref, reference; HR, hazard ratio; CI, confidence interval; GCS, Glasgow Coma Score; In age subgroup: adjusted gender, insurance status, urine output, acute kidney injury (AKI), diastolic blood pressure (DBP), temperature, Sequential Organ Failure Assessment (SOFA), Charlson comorbidity index (CCI), GCS, Simplified Acute Physiology Score II (SAPSII), white blood cell (WBC), hematocrit, hemoglobin, red cell distribution width (RDW), estimated glomerular filtration rate (eGFR), blood urea nitrogen (BUN), glucose, sodium, vasopressor, mechanical ventilation, and mannitol. In gender subgroup: adjusted age, insurance status, urine output, AKI, DBP, temperature, SOFA, CCI, GCS, SAPSII, WBC, hematocrit, hemoglobin, RDW, eGFR, BUN, glucose, sodium, vasopressor, mechanical ventilation, and mannitol. In GCS subgroup: adjusted age, gender, insurance status, urine output, AKI, DBP, temperature, SOFA, CCI, SAPSII, WBC, hematocrit, hemoglobin, RDW, eGFR, BUN, glucose, sodium, vasopressor, mechanical ventilation, and mannitol.

## Discussion

This retrospective study investigated the therapeutic potential of thiamine supplementation in TBI management using data from the MIMIC-IV database. Our analysis revealed a significant association between thiamine administration and reduced in-hospital mortality risk among critically ill TBI patients (HR = 0.46, 95% CI: 0.32–0.67, *p* < 0.001). Notably, this mortality reduction remained statistically significant after rigorous adjustment for injury severity, comorbidities, and treatment covariates through multivariable logistic regression modeling (HR = 0.66, 95% CI: 0.45–0.98, *p* = 0.037). These findings suggest that this readily available micronutrient intervention, characterized by its favorable safety profile and low cost, may represent a novel neuroprotective strategy for improving clinical outcomes in TBI populations requiring intensive care.

The water-soluble vitamin thiamine, commonly known as vitamin B1, is essential for glucose metabolism as its bioactive form, thiamine pyrophosphate, acts as essential co-enzyme (17). Total thiamine levels (essentially TPP) are much lower in humans than other species with a normal steady-state whole body store of approximately 30 mg (18). The Recommended Daily Allowance (RDA) suggests the thiamine dose for adults as 1.1–1.2 mg/day (19). Critically ill patients with TBI often experience exacerbated thiamine deficiency due to heightened metabolic demands, insufficient supplementation, and increased urinary excretion (20). Recently, numerous published studies have suggested the beneficial effects of thiamine supplementation on the prognosis of patients (21, 22). For example, in a cross-sectional study from Korea, thiamine intake was found to be critically associated with lower risks of hypertension, myocardial infarction or angina, type 2 diabetes, depression and dyslipidemia after adjusting all potential confounders (23). The provision of thiamine after cardiac arrest improved neurological outcome and 10 days survival in a mouse model (24). In addition, receiving thiamine supplementation was found to have multiple benefits for patients with ventilator-associated pneumonia, including enhancing energy restoration, reducing the likelihood of certain complications, alleviating oxidative stress, and exerting an anti-inflammatory effect (25). These findings hold significant implications for improving patient prognosis and survival rates. Nevertheless, there are few data to support relationship between the thiamine supplementation and in-hospital mortality of TBI patients.

After adjusting multiple confounding factors, our study showed that TBI patients receiving thiamine use have a lower risk of in-hospital mortality, which provided a new perspective to develop therapeutic strategies for TBI. The exact biological mechanisms by which thiamine could exert protective effects for patients with TBI remain unclear. The pivotal role of thiamine in mitochondrial energy metabolism and its involvement in various metabolic processes within mitochondria and peroxisomes *in vivo*, while also conferring cellular resistance against oxidative stress [16, 17]. Mitochondrial dysfunction, oxidative stress and inflammation contribute to the ongoing brain injury and cellular death (7). The administration of thiamine has been demonstrated to mitigate histological brain injury, enhance mitochondrial dynamics, and restore mitochondrial PDH complex activity through activation of the mitochondrial PDH complex (24). Thiamine deficiency has also been proposed as a possible cause of serious damage to brain regions (26). A systematic review suggested that thiamine supplementation may have a positive effect on the delay and prevention of cognitive decline (27). A study conducted on animals demonstrated that thiamine administration can potentially enhance brain homeostatic mechanisms and physiological fitness (28). Previous studies have also described the effect of vitamin for TBI (29, 30). TBI resulted in the manifestation of neurological deficits, accompanied by cerebral edema, disruption of the blood–brain barrier, and an inflammatory response (31). According to the above literature, Thiamine, in its active form TPP, plays a pivotal role in central nervous system energy metabolism by supporting oxidative decarboxylation reactions critical for ATP production. Additionally, it modulates neurotransmitter synthesis and myelin lipid metabolism, both of which are essential for maintaining neuronal integrity and signal transduction. In addition, our study revealed significant associations between thiamine administration and in-hospital mortality among elderly patients (≥65 years), male individuals, and those with severe TBI. These results also suggest that thiamine supplementation may confer prognostic benefits within this specific population.

However, this study has several limitations. First, due to its nature of single-center retrospective design, selection bias was inevitable. Second, although multivariate adjustment was employed, certain potential confounding factors that could impact the mortality of TBI patients, such as brain imaging data, administration time of thiamine and dosage of thiamine, were unobtainable in this MIMIC-IV database. Finally, this study was solely performed utilized data of patients admitted to the ICU, further investigation is warranted to explore the potential impact of thiamine on the prognosis of TBI patients in general wards.

## Conclusion

In short, thiamine administration may reduce in-hospital mortality for patients with TBI admitted to the ICU. Nevertheless, further randomized controlled trials with large sample sizes are needed to confirm the efficacy of thiamine in TBI treatment.

## Data Availability

Publicly available datasets were analyzed in this study. This data can be found at: MIMIC-IV database, https://mimic.physionet.org/iv/.
