# Supplementary material for: Thiamine administration and in-hospital mortality in patients with traumatic brain injury: analysis of the MIMIC-IV database
Source: Front Neurol. 2025 Apr 24;16:1448439. doi: 10.3389/fneur.2025.1448439 (PMC12058809; doi:10.3389/fneur.2025.1448439)
Supplement: Supplementary file 1 [file Table_1.docx]

**Supplementary Table 1 Sensitivity analysis on the data sets before and after interpolation**

| Variables | Groups | | Statistics | *P* |
| --- | --- | --- | --- | --- |
|  | After interpolation | Before interpolation |  |  |
| Heart rate (bmp), Mean ± SD | 84.83 ± 18.53 | 84.87 ± 18.52 | t=-0.06 | 0.949 |
| SBP (mmHg), Mean ± SD | 132.73 ± 22.87 | 132.78 ± 22.87 | t=-0.07 | 0.948 |
| DBP (mmHg), Mean ± SD | 72.41 ± 16.98 | 72.42 ± 16.99 | t=-0.01 | 0.988 |
| Respiratory rate (insp/min), Mean ± SD | 18.52 ± 5.17 | 18.57 ± 5.17 | t=-0.26 | 0.794 |
| Temperature (Deg.C), Mean ± SD | 36.80 ± 0.80 | 36.80 ± 0.80 | t=-0.09 | 0.929 |
| SPO_2_ (%), Mean ± SD | 97.52 ± 3.62 | 97.51 ± 3.63 | t=0.02 | 0.982 |
| Calcium (mg/dL), Mean ± SD | 8.09 ± 1.48 | 8.08 ± 1.49 | t=0.22 | 0.826 |
| eGFR (mL/min/1.73m^2^), Mean ± SD | 95.20 ± 14.58 | 95.22 ± 14.59 | t=-0.03 | 0.975 |
| Hemoglobin (g/dL), Mean ± SD | 11.40 ± 2.03 | 11.39 ± 2.03 | t=0.09 | 0.925 |
| Hematocrit (%), Mean ± SD | 34.20 ± 5.78 | 34.18 ± 5.78 | t=0.06 | 0.950 |
| Glucose (mg/dL), M (Q_1_, Q_3_) | 123.00 (102.00, 154.00) | 123.00 (102.00, 154.00) | Z=0.090 | 0.929 |
| BUN (mg/dL), M (Q_1_, Q_3_) | 15.00 (11.00, 22.00) | 15.00 (11.00, 22.00) | Z=0.010 | 0.992 |
| Platelet (K/uL), M (Q_1_, Q_3_) | 189.00 (146.00, 242.00) | 188.50 (146.00, 242.00) | Z=-0.044 | 0.965 |
| WBC (K/uL), M (Q_1_, Q_3_) | 10.20 (7.50, 13.20) | 10.20 (7.50, 13.20) | Z=-0.060 | 0.952 |
| RDW (%), Mean ± SD | 14.23 ± 1.90 | 14.23 ± 1.91 | t=-0.11 | 0.915 |
| Sodium (mEq/L), Mean ± SD | 138.80 ± 4.90 | 138.83 ± 4.87 | t=-0.18 | 0.860 |
| Potassium (mEq/L), Mean ± SD | 4.07 ± 0.71 | 4.07 ± 0.71 | t=0.04 | 0.971 |
| Chloride (mEq/L), Mean ± SD | 103.67 ± 5.70 | 103.69 ± 5.69 | t=-0.14 | 0.891 |
| Urine output (mL), M (Q_1_, Q_3_) | 1632.00 (1070.00, 2310.00) | 1630.00 (1070.00, 2325.00) | Z=0.015 | 0.988 |
| SOFA, M (Q_1_, Q_3_) | 1.00 (0.00, 1.00) | 1.00 (0.00, 1.00) | Z=-0.012 | 0.990 |
| GCS, Mean ± SD | 13.01 ± 2.69 | 13.01 ± 2.69 | t=0.02 | 0.980 |
| AKI, n (%) |  |  | χ^2^=0.000 | 0.989 |
| No | 739 (42.11) | 739 (42.13) |  |  |
| Yes | 1016 (57.89) | 1015 (57.87) |  |  |

SBP, systolic blood pressure; DBP, diastolic blood pressure; SPO_2_, pulse oxygen saturation; eGFR, estimated glomerular filtration rate; BUN, blood urea nitrogen; WBC, white blood cell; RDW, red cell distribution width; SOFA, Sequential Organ Failure Assessment; GCS, Glasgow Coma Score; AKI, acute kidney injury.

.

**Supplementary Table 2 Screening of confounding variables affecting in-hospital mortality**

| **Variables** | **HR (95%CI)** | ***P*** |
| --- | --- | --- |
| Age | 1.02 (1.02-1.03) | <0.001 |
| Gender |  |  |
| Female | Ref |  |
| Male | 0.74 (0.57-0.97) | 0.031 |
| Race |  |  |
| White | Ref |  |
| Black | 0.95 (0.52-1.72) | 0.861 |
| Other | 1.48 (1.12-1.95) | 0.005 |
| Insurance |  |  |
| Medicaid | Ref |  |
| Medicare | 1.76 (1.05-2.95) | 0.032 |
| Other | 0.89 (0.52-1.50) | 0.648 |
| ICU type |  |  |
| MICU | Ref |  |
| SICU | 0.80 (0.47-1.36) | 0.412 |
| TSICU | 0.63 (0.38-1.04) | 0.071 |
| Other | 0.71 (0.41-1.23) | 0.221 |
| Urine output | 1.01 (1.01-1.01) | 0.003 |
| Hypertension |  |  |
| No | Ref |  |
| Yes | 1.21 (0.92-1.58) | 0.173 |
| Deterioration of neurological function |  |  |
| No | Ref |  |
| Yes | 0.81 (0.61-1.08) | 0.149 |
| AKI |  |  |
| No | Ref |  |
| Yes | 1.96 (1.40-2.76) | <0.001 |
| Heart rate | 1.00 (0.99-1.01) | 0.960 |
| SBP | 1.00 (0.99-1.00) | 0.572 |
| DBP | 0.99 (0.98-0.99) | 0.006 |
| Respiratory rate | 1.01 (0.98-1.03) | 0.574 |
| Temperature | 0.75 (0.67-0.84) | <0.001 |
| SPO_2_ | 1.01 (0.97-1.05) | 0.557 |
| SOFA | 1.21 (1.14-1.28) | <0.001 |
| CCI | 1.09 (1.02-1.15) | 0.006 |
| GCS | 0.91 (0.87-0.95) | <0.001 |
| SAPSII | 1.06 (1.05-1.07) | <0.001 |
| WBC | 1.02 (1.01-1.03) | <0.001 |
| Platelet | 1.00 (1.00-1.00) | 0.430 |
| Hematocrit | 0.97 (0.95-0.99) | 0.003 |
| Hemoglobin | 0.90 (0.84-0.95) | <0.001 |
| RDW | 1.20 (1.14-1.26) | <0.001 |
| eGFR | 0.98 (0.97-0.99) | <0.001 |
| BUN | 1.02 (1.01-1.02) | <0.001 |
| Glucose | 1.01 (1.01-1.01) | 0.008 |
| Calcium | 1.01 (0.93-1.10) | 0.767 |
| Sodium | 1.03 (1.01-1.06) | 0.031 |
| Potassium | 1.15 (0.98-1.36) | 0.089 |
| Chloride | 1.01 (0.99-1.04) | 0.186 |
| Vasopressors |  |  |
| No | Ref |  |
| Yes | 2.52 (1.89-3.35) | <0.001 |
| Mechanical ventilation |  |  |
| No | Ref |  |
| Yes | 2.18 (1.48-3.19) | <0.001 |
| Mannitol |  |  |
| No | Ref |  |
| Yes | 3.20 (2.07-4.93) | <0.001 |
| Diuretic |  |  |
| No | Ref |  |
| Yes | 1.31 (0.98-1.75) | 0.065 |
| Surgery |  |  |
| No | Ref |  |
| Yes | 0.84 (0.21-3.38) | 0.807 |

ICU, intensive care unit; MICU, medical intensive care unit; SICU, surgical intensive care unit; TSICU, trauma and surgical intensive care unit; AKI, acute kidney injury; SBP, systolic blood pressure; DBP, diastolic blood pressure; SPO_2_, pulse oxygen saturation; SOFA, Sequential Organ Failure Assessment; CCI, Charlson comorbidity index; GCS, Glasgow Coma Score; SAPSII, Simplified Acute Physiology Score II; WBC, white blood cell; RDW, red cell distribution width; eGFR, estimated glomerular filtration rate; BUN, blood urea nitrogen; Ref, reference; HR, hazard ratio; CI, confidence interval.
